# Examinations for the Doctorate in the University of Virginia

**Published:** 1847-04

**Authors:** 


### EXAMINATIONS FOR THE DOCTORATE IN THE UNIVERSITY OF VIRGINIA.

We have lately received from one of the professors of this University a printed sheet of questions submitted to the candidates for graduation in 1846. We presume the questions are put into the hands of the candidate to be answered forthwith in writing, without the assistance of books or friends. After traveling through them, we feel bound to admit that if no candidate graduated unless he answered the majority of the interrogatories correctly, the alumni of that school are better informed than those of the country generally. The questions are arranged under nine different heads,—Anatomy, Physiology, Surgery, Obstetrics, Chemistry, Materia Medica, Therapeutics, Practice of Medicine, and Medical Jurisprudence. As specimens of the whole, we extract the articles 'Surgery' and 'Practice of Medicine:'

"*Surgery.*—1. Give the symptoms, pathology and treatment of the shock produced by extensive burns.

2. After reaction has been some time established, and during the progress of the case, what internal organs are most liable to suffer, and in what manner ?

3. What is the third period of danger in burns of the third degree?

4. Describe the pathology of metastatic or consecutive abscess, stating the characters of the primary lesion and the signs on which the diagnosis is formed.

5. Give the diagnostic symptoms and the treatment of diffused arteritis, and state its most common effects where resolution is not obtained.

6. Give the pathology and diagnosis of spontaneous aneurisms and their treatment by Hunter's method.

7. Give the causes, signs and treatment of fracture of the clavicle;

8. Of fracture of the fibula near the malleolus.

9. Give the symptoms of iritis, and state the distinct indications of treatment, and the considerations on which they are founded.

10. Describe the common operation of lithotomy.

11. Give the differential diagnosis of the different tumors of the scrotum and testicle."

"*Practice of Medicine.*—Give the semiology, etiology, pathological anatomy and special pathology and therapeutics—1st, of diabetes mellitus; 2d, of acute muco-enteritis; 3d, of ascites; 4th, of pulmonary apoplexy; 5th, of phthisis pulmonalis; 6th, of phlegmasia alba dolens; 7th, of cerebral apoplexy; 8th, of eclampsia parturientium; 9th, of bilious remittent fever; and, 10th, of typhoid fever.

Give the general pathology of inflammation, dropsy, hemorrhage, tuberculosis, convulsions, and fever."

These are the shortest of the nine, and yet either of them would be a good day's work even for a professor.

